# Supplementary figures and images for: Neutralizing monoclonal antibodies improve biodistribution of intravenously administered oncolytic adenovirus in human CD46-transgenic mice
Source: PLoS One. 2025 Jun 25;20(6):e0326857. doi: 10.1371/journal.pone.0326857 (PMC12193558; doi:10.1371/journal.pone.0326857)

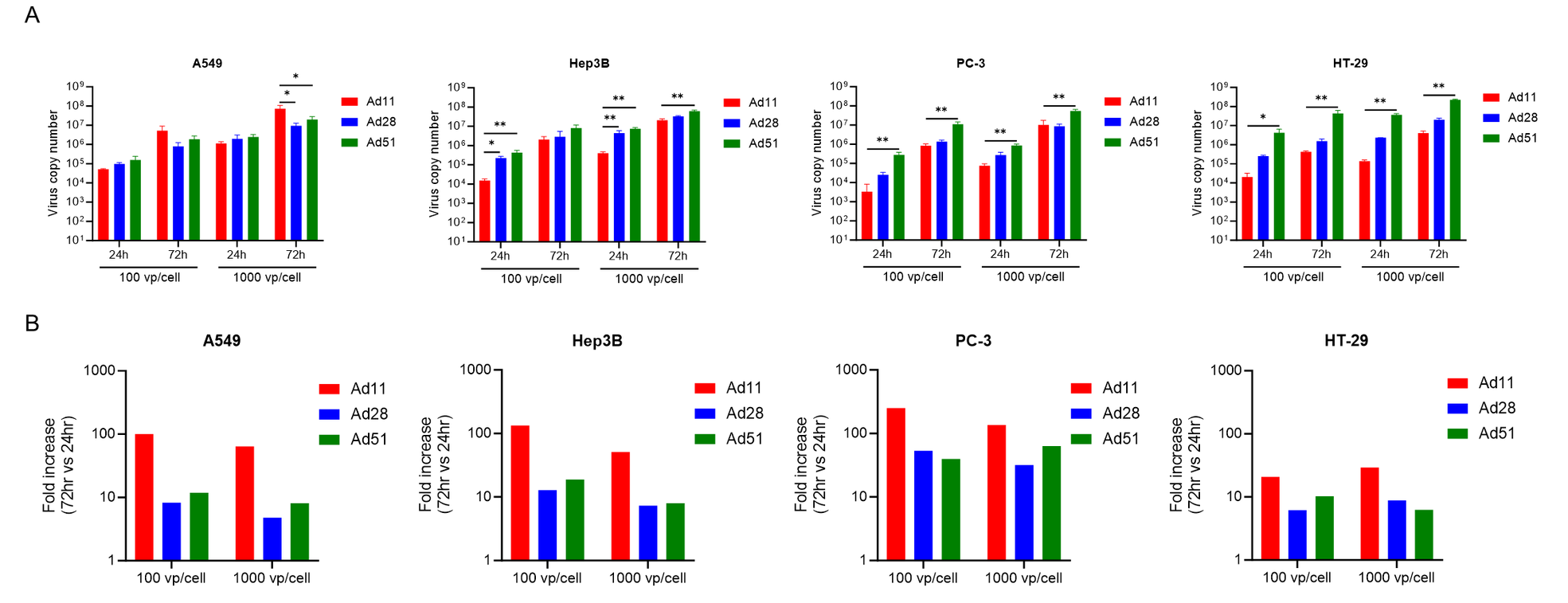

Supplement: S1 Fig — (A) Cells were infected with Ad11, Ad28, and Ad51 at 100 and 1000 vp/cell in vitro. Twenty-four and seventy-two hours after viral infection, viral genomic DNA was quantified by qPCR. The experiment was conducted in triplicate. Mean ± SD is shown. *P < 0.05 and **P < 0.01 compared with Ad11 by Dunnett’s multiple comparison test. (B) Time-dependent change in virus copy number between 24 and 72 hours. (TIF) [file pone.0326857.s001.tif]

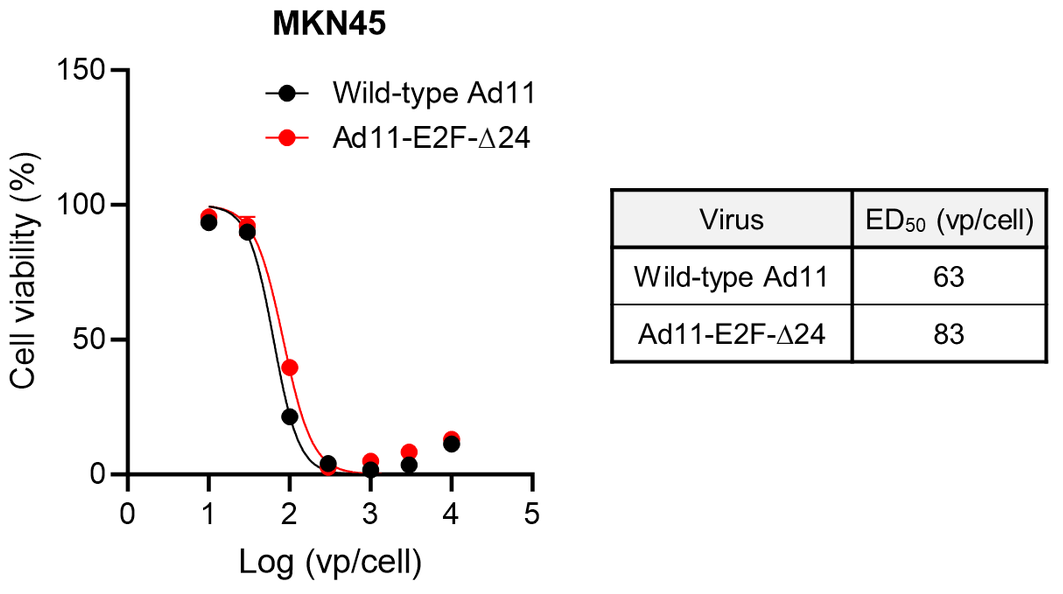

Supplement: S2 Fig — MKN45 was infected with Ad11 and Ad11-E2F-∆24 at different titers (vp/cell). Five days after viral infection, cytotoxicity was measured. The experiment was conducted in triplicate. Mean ± SD is shown. Table indicates the ED50 values for each cell line. (TIF) [file pone.0326857.s002.tif]

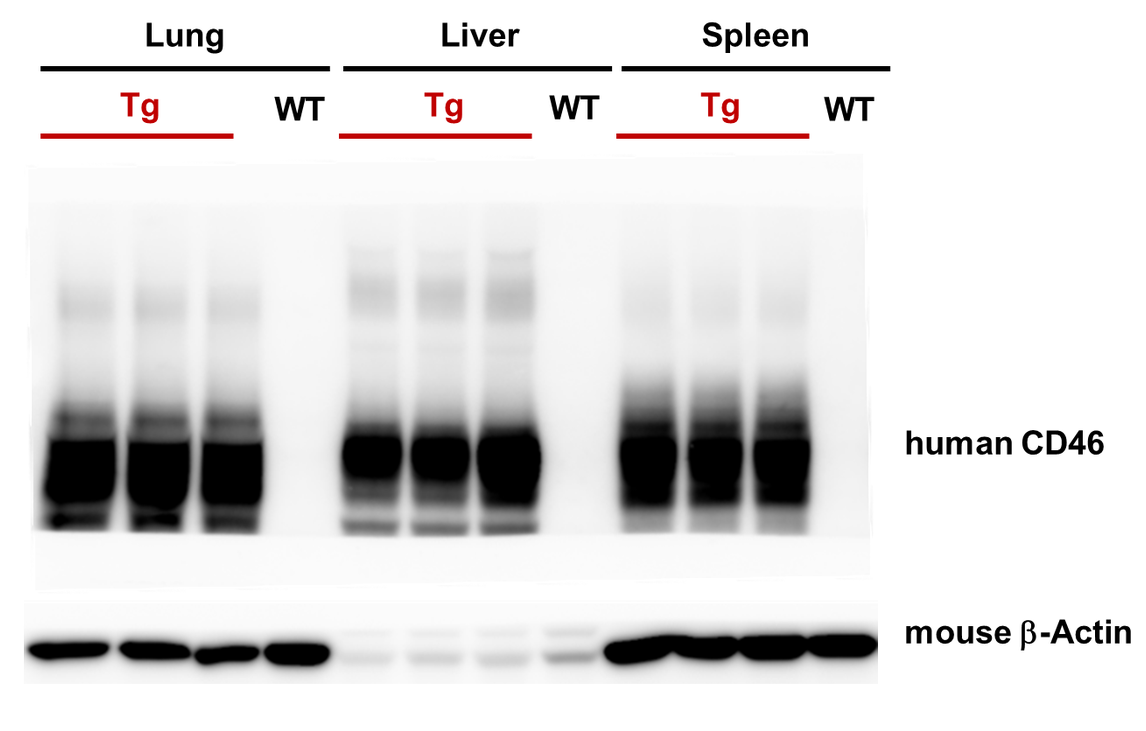

Supplement: S3 Fig — Homogenates of organs from hCD46-tg mice were subjected to western blotting analysis using the indicated antibodies. Homogenates of organs from Balb/c mice were used as a negative control. Human CD46-tg mice; Tg, Balb/c mice; WT. (TIF) [file pone.0326857.s003.tif]

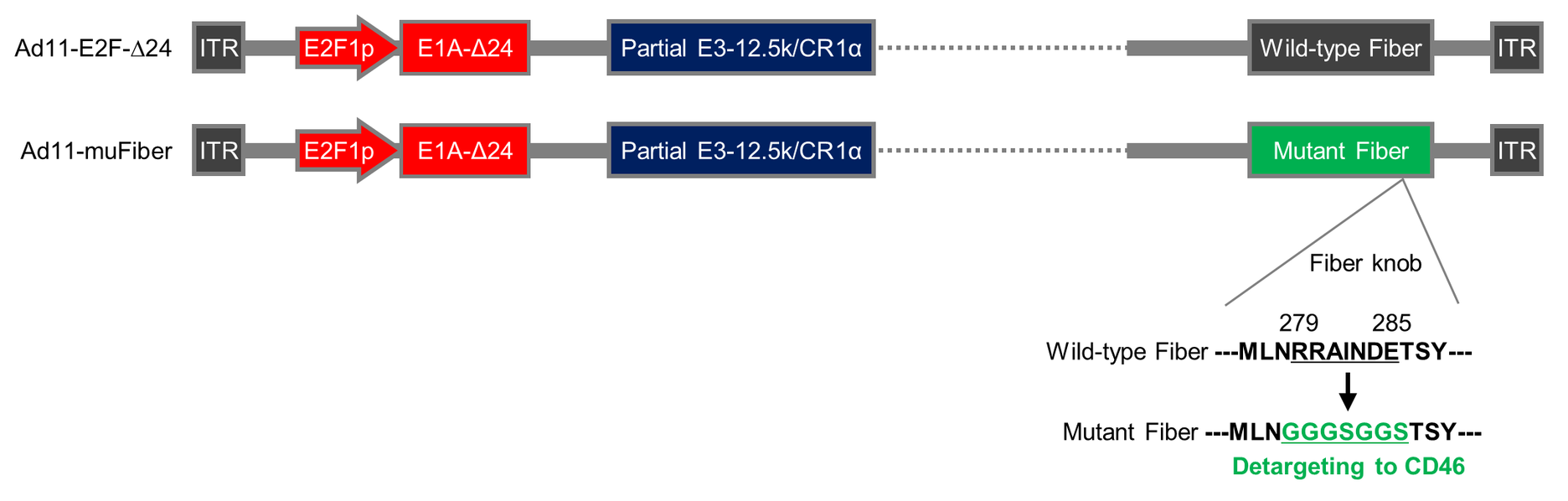

Supplement: S4 Fig — For de-targeting to CD46, the CD46-binding motif in the fiber knob of Ad11-E2F-∆24 was replaced with a GS linker. (TIF) [file pone.0326857.s004.tif]
