# Supplementary material for: Neutralizing monoclonal antibodies improve biodistribution of intravenously administered oncolytic adenovirus in human CD46-transgenic mice
Source: PLoS One. 2025 Jun 25;20(6):e0326857. doi: 10.1371/journal.pone.0326857 (PMC12193558; doi:10.1371/journal.pone.0326857)
Supplement: Attachment — Submitted filename: PONE_Review_12.24.docx [file pone.0326857.s006.docx]

- Are a* unique.

With the animal model in mind, it’s not clear which metastases would be implicated, lung metastases, brain?

Not sure one can say “the virus was infected.”?

It’s not clear in the narrative if it was DNA/RNA/etc.. that was performed using qPCR

It might be specified later, but it would be convenient to know which organs were collected.

What does conventional methods mean? I think that this section could be more detailed, the specificity of the monoclonal antibodies is at the core of the paper’s hypothesis of reducing toxicity, however, the authors provide little explanation as to how the antibodies were selected, and how inhibition of Ad11 was identified.

Which organs? Specify

Which proteins were measured here? It’s not clear.

Given the importance of the selection of which adenovirus would have the least affinity, there seems to be very little detail provided about the pre-exposure of the donors to adenovirus, with a number (n=5) that might not be representative of total population affinity characteristics.

In this context, it could be highlighted which receptor is used by Ad5.

A dot seems to be missing here.

In light of the results stipulated in the manuscript regarding the cytotoxicity Ad11, I would think it’s important to show statistical assessments here. Also, the number of days should be indicated on the graphs. The absence of a negative is also noted.

Within the same reasoning and considering the absence of any statistical tests that would reinforce this hypothesis, the strength of cytotoxicity cannot be evaluated.

No statistical tests have been performed to test the hypothesis regarding cytotoxicity in fibroblasts vs colorectal nor adenocarcinoma cell lines.

This statement has to be supported by statistical tests and there are no details about how this secretion was tested, through infection in vitro? There is a lack of details regarding the process of the experiment that led to this conclusion.

Absence of statistical testing to support the claims. Typo: Relative*. Axis title could be renamed to reflect what was measured, and the nature of the qPCR performed. The sample size seems small and would be limiting the statistical power. (n=3?) Also, it is not clear from the narrative why a smaller number of mice were used for Ad5?

These results depend mainly on the tested results on HEK293, while there is no mention of the choice for this cell line, instead of lung or ovarian cancer cell line that were mentioned previously.

In this graph, the is absence of non-specific control is noted, which would be needed to corroborate the effectiveness of 4G1. Were the results statistically analysed to determine significance? Is there any suggestion of dose-response to 4G1 that determines the dose-response relationship and antibody’s potency?

Statistical significance?

Incubation at 37°C and only an hour is not justified here, ideally, there would be a time-course study to indicate the optimal incubation time. Unless previously performed.

The authors show only mice that were treated, there are no control groups or non-specific antibodies.

This transient nature of the antibody binding needs to be investigated further in matters of reversibility and duration of effect.
