# Supplementary material for: Neutralizing monoclonal antibodies improve biodistribution of intravenously administered oncolytic adenovirus in human CD46-transgenic mice
Source: PLoS One. 2025 Jun 25;20(6):e0326857. doi: 10.1371/journal.pone.0326857 (PMC12193558; doi:10.1371/journal.pone.0326857)
Supplement: Attachment — Submitted filename: M.Hemmi_Response to Reviewers_May2025.docx [file pone.0326857.s008.docx]

**Response to Reviewer #2:**

Reviewer #2: General comments:

The authors have addressed most of the requests and issues suggested by the Reviewer during the revision, but there are few critical issues that persist after the initial revision process.

Thus, the manuscript is not suitable for publication in the current state.

Specific Comments:

1. In response to Q4 by Reviewer #2, the authors reported that the superior cell killing effect of Ad11 over Ad28 or Ad51 in Figure 2 did not correlate with their replication capacity based on the results from newly added Supplementary Figure S1. However, this conclusion made by the authors seems premature and inadequate, since the data clearly demonstrated that time-dependent change in virus copy number between 24 and 72 hours is notably higher in Ad11 compared to other two Ad serotypes in both A549 and Hep3B cells at all dose levels. Specifically, the fold change in viral titer in both A549 and Hep3B cells from 24h to 72h after inflection with Ad11 is nearly 100-fold (2 log increase), whereas the both Ad28 and Ad51 only exhibit around 10-fold (1 log) increase in viral quantity. These results would indicate that Ad11 generated nearly 10-fold more progeny virus over 48 h period than Ad28 or Ad51, suggesting more effective replication capacity. The lower level of Ad11 virus copy number compared to Ad28 or Ad51 in Hep3B cells at 72 hours after infection at 1,000 vp/cell were likely due to these cells being less susceptible to cellular internalization of Ad11 compared to the other two adenovirus serotypes, as suggested by nearly 10-fold lower viral quantity being detected at early time after the infection (24 h). The difference in final viral quantity at 72 hours after the infection by Ad11 versus Ad28/Ad51 is markedly lower than those observed at 24 hours after infection. Due to these reasons, it is premature to conclude that superior cytolytic effect of Ad11 was not achieved through higher replication efficiency.

Thus, additional experiments evaluating viral replication capacity in few other cancer cell lines and cellular uptake efficiency of Ad11, Ad28, and Ad51 would be able to more clearly elucidate whether the superior cytotoxicity of Ad11 was either replication-dependent or -independent.

*Response:*

According to the Reviewer’s suggestion, we further evaluated virus replication in PC-3 and HT-29 cells. Our results showed that Ad11 exhibited consistently higher increase in viral copy numbers across all tested cell lines (Supplementary figure 1). These findings suggests that the enhanced virus replication may contribute to the superior cytotoxicity of Ad11, whereas other mechanisms may also be involved in its potent killing activity, as reported by Wong et al. (Molecular Therapy, 2012). The results have been incorporated, and the conclusion has been revised accordingly.

2. Authors' response to Reviewer #2's Q9 that requested for safety and efficacy evaluation of their oncolytic adenovirus candidate in hCD46tg mice model is not adequate. Authors have stated "poor susceptibility of murine cells to Ad11" as the main reason for not performing safety assessment in mouse models, but there are several issues with this response.

Firstly, the main scope of this study was to identify alternative Ad serotype that is more suitable for intravenous administration application than Ad5, as authors noted that nonspecific liver sequestration and toxicity along with hemolytic side effect of Ad5 as major limitations of Ad5 for intravenous administration. As authors have highlighted several safety concerns regarding Ad5 as an intravenous administrable therapeutic platform, even a preliminary safety assessment of their newly developed intravenously administrable oncolytic adenovirus platform should be provided.

Secondly, authors have noted that monkeys are a much better model for safety analysis, and we agree with this point. However, no safety assessment data in monkeys have been provided by the authors during the revision, and clinical relevance of mice model for preliminary safety assessment of intravenously administered adenovirus should not be completely overlooked. For example, it is extremely common for reports investigating intravenously administered Ad5-based therapy to include safety analysis data from mice before they advance to more clinically relevant animal models like Syrian hamsters or Monkeys (Commun Biol. 2024 Sep 13;7(1):1132. Mol Ther Oncolytics. 2017 Oct 26:7:76-85. J Immunother Cancer. 2021 Nov 9;9(11):e003254.), despite the mice model being poorly permissive and susceptible to Ad5 infection in similar manner to Ad11. Additionally, it is important to note that many of the toxicities observed in mice models were also recapitulated in human patients (e.g., immune-related adverse events and liver toxicity).

Lastly, authors have chosen to utilize mouse IL-12 as therapeutic gene of choice in their oncolytic adenovirus candidate and assessment of its effect would be most relevant in mice models rather than monkey or humanized models. As IL12, a potent pro-inflammatory cytokine, could exacerbate immune-related adverse events after systemic administration, it is essential to evaluate whether this transgene can be safely expressed without significant off-target toxicity after intravenous administration by oncolytic adenovirus.

In absence of presentable safety analysis data from monkey, it is essential for authors to investigate preliminary safety profile of their IL12-expressing oncolytic adenovirus after systemic administration to better support their claim that Ad11-based oncolytic virotherapy is a more suitable candidate for intravenous therapy than conventional Ad5.

*Response:*

We appreciate insightful suggestions from the Reviewer. Based on previous reports investigating the safety of intravenously administered Ad5, we assessed the replication of Ad11 in cells derived from mouse, hamster, and monkey. Our results showed that Ad11 did not replicate in mouse and hamster cell lines (please find figures below), that is the reason why we did not pursue mouse models previously, although we acknowledge their clinical relevance for safety assessment.

In this revision, we evaluated the safety of wild-type Ad5, wild-type Ad11, and recombinant Ad11s in mice. Intravenous administration of Ad5 resulted in acute body weight loss and elevation of plasma AST and ALT levels. Intravenous administration of Ad11s did not increase AST or ALT levels, while they induced a transient body weight loss, which resolved within a few days (Figure 4D and 4E).
